# Daytime use of general practice and use of the Out-of-Hours Primary Care Service for patients with chronic disease: a cohort study

**DOI:** 10.1186/1471-2296-15-156

**Published:** 2014-09-20

**Authors:** Lone Flarup, Grete Moth, Morten Bondo Christensen, Mogens Vestergaard, Frede Olesen, Peter Vedsted

**Affiliations:** Department of Public Health, Research Unit for General Practice, Aarhus University, DK-Bartholins Allé 8000 Aarhus C, Aarhus, Denmark; Department of Public Health, Danish Research Centre for Cancer Diagnosis in Primary Care (CaP), Aarhus University, Aarhus, Denmark; Department of Public Health, Section for General Medical Practice, Aarhus University, Aarhus, Denmark

## Abstract

**Background:**

The importance of proactive chronic care has become increasingly evident. Yet, it is unknown whether the use of general practice (GP) during daytime may affect the use of Out-of-Hours (OOH) Primary Care Service for people with chronic disease. We aimed to analyse the association between use of daytime general practice (GP) and use of OOH services for heart disease, lung disease, diabetes, psychiatric disease, or cancer. In particular, we intended to study the association between OOH contacts due to chronic disease exacerbation and recent use of daytime GP.

**Methods:**

Data comprised a random sample of contacts to the OOH services (‘LV-KOS2011’). Included patients were categorised into the following chronic diseases: heart disease, lung disease, diabetes, psychiatric disease, or cancer. Information on face-to-face contacts to daytime GP was obtained from the Danish National Health Insurance Service Registry and information about exacerbation or new episodes from the LVKOS2011 survey. Associations between number of regular daytime consultations and annual follow-up consultations during one, three, six, and 12 months prior to index contacts, and outcomes of interest were estimated by using logistic regression.

**Results:**

In total, 11,897 patients aged ≥ 18 years were included. Of these, 2,665 patients (22.4%) were identified with one of the five selected chronic diseases; 673 patients (5.7%) had two or more. A higher odds ratio (OR) for exacerbation as reason for encounter (RFE) at the index contact was observed among patients with psychiatric disease (OR = 2.15) and cancer (OR = 2.17) than among other patients for ≥2 daytime recent contacts. When receiving an annual follow-up, exacerbation OR at index contact lowered for patients with lung disease (OR = 0.68), psychiatric disease (OR = 0.42), or ≥2 diseases (OR = 0.61).

**Conclusion:**

Recent and frequent use of daytime GP for patients with the selected chronic diseases was associated with contacts to the OOH services due to exacerbation. These findings indicate that the most severely chronically ill patients tend to make more use of general practice. The provision of an annual follow-up daytime GP consultation may indicate a lower risk of contacting OOH due to exacerbation.

## Background

Proactive and comprehensive health care for people with chronic diseases has recently received increased focus. According to the World Health Organisation (WHO), chronic diseases are the major cause of death and disability worldwide [1]. The proportion of people living with chronic diseases is currently increasing due to modern life style in combination with longer life expectancy in all western countries, and these patients are among the highest consumers of all healthcare resources [2, 3]. Primary care has become extremely important in providing high quality and coordinated healthcare for people with chronic diseases [4, 5].

In the Danish healthcare system, general practice is gatekeeper for the rest of the healthcare system and thereby the first in line to handle both daytime healthcare and the out-of-hours services (OOH) [6, 7]. Thus, general practice holds an important position as the place that all patients contact to get medical advice, diagnosis, treatment and follow-up regardless if they have a chronic disease or not. General practitioners (GPs) thereby also serve as gatekeepers to an increasingly specialised medical technology for those patients who need a intensively medical attention or hospital care [4].

Care of chronic diseases should optimally be performed during daytime and not during out-of-hours, where the reason for encounter (RFE) should be an acute presentation. It would be expected that the more people with chronic disease use daytime general practice, the more they would also use the OOH services due to their general high need of medical attention at all hours [8]. Nevertheless, one could hypothesize that regular disease monitoring by the GP during daytime may result in a lower need for OOH care due to exacerbations for patients with chronic diseases than for patients with no or only little contact to their GP. Associations between daytime GP use and OOH use for people with different chronic diseases remains unknown, but more knowledge about this area is needed as e.g. implementation of more proactive daytime care targeting patients with chronic diseases has considerable implications for future health-care planning.

With this study, we aimed to analyse associations between use of daytime general practice and contact to the OOH Primary Care Service for patients with heart disease, lung disease, diabetes, severe psychiatric disease or cancer. Furthermore, we aimed to analyse whether contact to the OOH services due to an exacerbation of a chronic disease was associated with recent face-to-face contacts with the daytime GP.

## Methods

### Design and setting

The study was based on information on 21,457 randomly sampled patients contacting the OOH services in the Central Denmark Region, which covers approximately 1.3 million residents [9]. These data were originally collected for the ‘LV-KOS2011’ survey (LV-KOS); a large and comprehensive Danish cross-sectional study on disease patterns in the OOH Primary Care Service during a 12-month period in 2010-2011. The survey was carried out by means of pop-up questionnaires integrated into the existing electronic OOH patient administration system. The OOH-GPs were, for instance, asked whether the RFE in question was a new event or an exacerbation of an already diagnosed chronic disease. The GPs included three independent types of contacts to OOH; *telephone consultations*, *clinical consultations*, or *home visits*. The pop-up frequency was adjusted to the GP workload and the sample size required for valid research results; 1 in every 10 telephone contacts, 1 in every 3 clinical consultations, and every home visit. For each of the three types of contact, registered contacts were highly representative of all contacts to the OOH services in the region during the study period, and the participating GPs were representative of all GPs working in the OOH services during the same period [10].

### Study population

From the data obtained in the LV-KOS survey, we included patients aged 18 years or older. We excluded patients without a unique Danish civil registration (CPR) number, assigned to all Danish citizens [11]. The cohort was categorised with reference to chronic disease defined as heart disease, lung disease, psychiatric disease, diabetes, and cancer by linking the unique Danish CPR [11] with Danish registry data [12].

### Data

Eligible patients with the five chronic diseases were identified by using the International Classification Of Diseases (ICD-10) [13] or by a combination of ICD-10 codes, data on prescribed medication according to the Anatomical Therapeutic Chemical (ATC) Classification System [14, 15], and data on health services provided in general practice [16] (Table 1). The registry data used for the identification was obtained for the period of 1 January 2005 until 30 days before inclusion in the LV-KOS study (index date).

**Table 1:** **Sources and codes for identification of chronically ill patients**

	National registries in Denmark	Variables
**Heart disease**	Danish National Registry of Patients	ICD-10: I20, I21
**Lung disease**	Danish National Registry of Patients	ICD-10: J40-J47, or J96
	Danish National Health Service Register	Service codes: 7113, 7121 (spirometries)
	Danish National Prescription Registry	ATC codes: R03AC, R03AK, R03BA, R03BB, R03CC, R03DA, R03DC, V03AN01 (oxygen)
**Diabetes**	Danish National Diabetes Register	Civil registration numbers (CPR) corresponding registry data
**Psychiatric disease**	Danish Psychiatric Central Registry	ICD-10: F20, F25, F30, F31
**Cancer**	Danish Cancer Registry	ICD-10: C00-C97 (excl. C44)

Patients with heart disease were defined as patients with unstable angina (ICD-10 I20.0) and post myocardial infarction (ICD-10 I21), and these patients were identified through the Danish National Patient Registry [17]. Patients with diabetes were identified if registered in the National Diabetes Registry [18]. Psychiatric disease was defined as schizophrenia (ICD-10 F20), and schizophrenia and schizoaffective disorders (ICD-10 codes F25), and bipolar affective disorder (ICD-10 codes F30 and F31), and patients were identified in the Danish Psychiatric Central Registry [19]. Patients with cancer were identified if registered with ICD-10 C00-C97, excluding C44 (non-melanoma skin cancers) in the Danish Cancer Registry [20]. Patients with lung disease were identified according to a validated algorithm [21]; ICD-10 J40-J47 or J96 (in the Danish National Patient Registry), *or* redemption of at least two prescriptions with ATC codes R03AC, R03AK, R03BA, R03BB, R03CC, and R03DA, R03DC (all including subcodes) within the past 12 months, *or* oxygen treatment with the ATC code V03AN01 (according to the Danish National Prescription Registry [14]), *or* at least two spirometries with service codes 7113 or 7121 within the past 12 months (the Danish National Health Service Registry [16]). A subgroup of patients was identified by two or more of the five diseases designated as ‘2 + diagnosed’. Patients with more than one of the five chronic diseases were included in the numerator for each specific disease.

Information on face-to-face contacts during daytime general practice (Table 2) was obtained from the Danish National Health Service Register during the 12-month period prior to the index date.

**Table 2:** **Remuneration codes used for analysis of provided daytime face-to-face consultations in the Danish general practice**

0101	Clinical consultation
0106	Preventive consultation (from 1 April 2011 included in 0101)
0107	Annual follow-up, annual follow-up-consultation for diabetes
0411-0461, 0491	Home visit
2304	Preventive consultation as annual follow-up (from 1 April 2011 included in 0120)
2305	Home visit regarding status of resources and need for care for elderly patients
0120	Preventive annual medical care consultation (from 1 April 2011)
0121	Annual medical care consultation for elderly patients (from 1 April 2011)
6101	Conversational therapy

From the LV-KOS survey, we obtained information as to whether the registered RFE was due to an exacerbation of an already diagnosed chronic disease or due to a new health problem.

### Statistical analyses

Descriptive analyses were performed for each of the five chronic-disease patient groups and for the group consisting of 2 + diagnosed patients. The study population was divided according to the LV-KOS survey information on whether the RFE was due to an exacerbation of previously diagnosed chronic disease or of a new health problem. Due to the sampling method (one in ten telephone contacts, one in three clinical consultations, and every home visit), we made weighted analyses using sample weights defined as the reciprocal to the sampling fraction when reporting on all contacts [22].

For each of the five patient groups, we calculated mean number of daytime contacts during one, three, six, and 12 months prior to the index date, proportions of patients in contact with daytime general practice, and proportions of patients with a scheduled and recommended annual follow-up at their own GP for the chronic condition. Continuous data were presented as means with corresponding 95% confidence intervals (95% CI), and proportions were presented in numbers with corresponding weighted percentages. Data on contacts to daytime general practice was analysed as categorical variables in weighted multiple logistic regressions presented as odds ratios (ORs) with the corresponding 95% CI. The ORs for RFEs due to exacerbation were calculated for use of daytime general practice 30 days prior to the index date and for annual follow-up, and were adjusted for age, gender, and type of LV-KOS contact. Adjustments for clustering by practice were made using robust variance estimates. Data were analysed using STATA /MP 12.1 (StataCorp LP, College Station, TX, USA).

### Ethics approvals

The project was approved by the Danish Data Protection Agency (J.no. 2011-41-6365). According to Danish law, approval from the ethical committee was not needed as the study did not include biomedical intervention.

## Results

From the LV-KOS population of 12,381 patients, the study population for the present study comprised 11,897 unique patients aged 18 years or older (Table 3). We thereby excluded 484 patients without a Danish civil registration number. Of the study population, 2,665 patients (22.4%) had one of the five chronic diseases selected for this study; 673 patients (5.7%) had two or more of the five chronic diseases. Except for diabetes, more women than men were identified with one of the chronic diseases for contacts due to exacerbation. The prevalence of chronic disease increased with age for all five chronic diseases selected for this study, except for psychiatric disease.

**Table 3:** **Distribution of study population according to gender, age, and exacerbation or new health problem** ^**1**^

	Heart disease N = 494 (100%)		Lung disease N = 210 (100%)		Diabetes N = 1,527 (100%)		Psychiatric disease N = 355(100%)		Cancer N = 1,491 (100%)		2+ diagnosed N = 673 (100%)		Remaining population N = 8,554 (100%)	
**Exacerbation** (n (%))	n = 152 (30.8)		n = 83 (39.5)		n = 381 (25.0)		n = 131 (36.9)		n = 336 (22.5)		n = 205 (30.5)		n = 1,213 (14.2)	
**Gender** (n (%^2^))														
Men	77	(46.3)	34	(48.9)	182	(47.8)	59	(50.0)	142	(39.9)	96	(50.0)	537	(42.4)
Women	75	(53.7)	49	(51.1)	199	(52.2)	72	(50.0)	194	(60.1)	109	(50.0)	676	(57.6)
Total	152	(100.0)	83	(100.0)		(100.0)	131	(100.0)	336	(100.0)	205	(100.0)	1,213	(100.0)
**Age groups in years** (n (%^2^))														
18-40	3	(6.7)	1	(0.5)	14	(6.6)	43	(27.0)	21	(11.6)	3	(0.5)	394	(44.1)
41-60	29	(20.5)	15	(26.4)	98	(29.6)	56	(44.0)	67	(24.5)	48	(26.0)	364	(29.9)
61-75	54	(36.4)	32	(39.0)	132	(34.0)	30	(28.5)	112	(34.0)	75	(28.4)	233	(13.4)
+75	66	(36.4)	35	(34.1)	137	(29.8)	2	(0.4)	136	(29.9)	79	(35.0)	222	(12.7)
Total	152	(100.0)	83	(100.0)	381	(100.0)	131	(100.0)	336	(100.0)	205	(100.0)	1,213	(100.0)
**New health problem** (n (%))	n = 342 (69.2)		n = 127 (60.5)		n = 1,146 (75.0)		n = 224 (63.1)		n = 1,155 (77.5)		n = 468 (69.5)		n = 7,341 (82.6)	
**Gender** (n (%^2^))														
Men	202	(44.1)	60	(47.5)	588	(50.1)	93	(40.7)	436	(31.9)	222	(43.7)	3,317	(43.0)
Women	140	(55.9)	67	(51.5)	558	(49.9)	131	(59.3)	719	(68.1)	246	(56.3)	4,024	(57.0)
Total	342	(100.0)	127	(100.0)	1,146	(100.0)	224	(100.0)	1,155	(100.0)	468	(100.0)	7,341	(100.0)
**Age groups in years** (n (%^2^))														
18-40	6	(4.0)	4	(4.2)	117	(17.2)	83	(43.8)	114	(18.5)	21	(0.9)	3,683	(57.6)
41-60	55	(22.1)	20	(17.8)	262	(28.7)	93	(40.1)	214	(24.1)	80	(26.1)	1,851	(25.1)
61-75	105	(31.2)	46	(40.9)	351	(28.9)	35	(14.0)	335	(28.1)	157	(32.1)	851	(8.0)
+75	176	(42.7)	57	(37.0)	416	(25.2)	13	(2.1)	462	(29.3)	210	(32.5)	956	(9.3)
Total	342	(100.0)	127	(100.0)	1,146	(100.0)	224	(100.0)	1,155	(100.0)		(100.0)	7,341	(100.0)

^1^Patients with more than one of the five chronic diseases were included in the numerator for each specific disease.

^2^Distribution-related weighted percentage for contacts.

At least one third of the patients with heart disease, lung disease, or psychiatric disease who had a face-to-face contact during daytime with their GP within 30 days prior to the index date of the OOH contact were registered with an exacerbation, while this was true for only approximately one in four of the patients with diabetes or cancer (Table 4). A low proportion of patients with chronic disease had an annual follow-up (20% or less), except for patients with diabetes for whom the proportion was 26%. The higher mean numbers of face-to-face GP daytime contacts for patients with an index contact due to exacerbation revealed that this group of patients tended to have higher use of daytime GP services compared to the remaining population without any of the five selected chronic diseases (Table 4).

**Table 4:** **Use of general practice during 30 days prior to the index OOH contact according to reason for encounter** ^**1, 2**^

	Heart disease N = 494		Lung disease N = 210		Diabetes N = 1,527		Psychiatric disease N = 355		Cancer N = 1,491		2+ diagnosed N = 673		Remaining population N = 5,894	
**Proportion of patients with a daytime face-to-face GP contact during 30 days prior to the index date** (n (%^3^))														
Exacerbation (index contact)	115	(31.2)	55	(38.9)	273	(25.9)	74	(33.0)	234	(23.9)	148	(30.4)	692	(14.6)
New health problem (index contact)	205	(68.8)	81	(61.4)	719	(74.1)	118	(67.0)	679	(76.1)	308	(69.6)	3,152	(85.4)
Total	320	(100.0)	136	(100.0)	992	(100.0)	192	(100.0)	913	(100.0)	456	(100.0)	3,851	(100.0)
**Proportion of patients provided an annual follow-up during the past 12 month** (n (%^3^))														
Exacerbation (index contact)	32	(23.1)	12	(27.7)	97	(23.7)	16	(21.0)	56	(20.1)	51	(20.4)	140	(17.5)
New health problem	68	(76.9)	25	(72.3)	305	(76.3)	22	(79.0)	182	(79.9)	113	(79.6)	486	(82.5)
Total	100	(100.0)	37	(100.0)	402	(100.0)	38	(100.0)	238	(100.0)	164	(100.0)	626	(100.0)
**Mean number of daytime face-to-face GP contacts according to time before index date** (n (%))														
Index contact due to exacerbation:														
1 month	1.5	(1.1;1.4)	1.4	(1.1;1.7)	1.8	(1.3;2.2)	1.8	(1.1;2.9)	1.6	(1.3;1.8)	2.0	(1.2;2.7)	0.7	(0.6;0.7)
3 months	3.7	(3.1;4.4)	3.6	(2.9;4.3)	4.7	(3.5;5.8)	5.3	(3.0;7.7)	3.4	(3.0;3.8)	5.0	(3.1;7.0)	2.5	(2.3;2.8)
6 months	7.2	(5.9;8.4)	7.0	(5.7;7.7)	8.8	(6.5;11.0)	10.1	(5.7;14.6)	6.3	(5.6;7.1)	9.7	(5.9;13.4)	4.7	(4.2;5.0)
12 months	13.1	(10.9;15.4)	12.5	(10.0;15.0)	16.5	(12.2;20.8)	18.9	(10.3;27.6)	11.1	(9.8;12.4)	18.5	(10.9;26.1)	8.6	(7.8;9.3)
Index contact due to new health problem:														
1 month	0.8	(0.5;0.8)	0.8	(0.7;0.9)	0.7	(0.6;0.8)	0.8	(0.7;0.9)	0.7	(0.7;0.8)	1.4	(1.2;1.7)	0.8	(0.7;0.8)
3 months	3.3	(2.8;3.8)	2.0	(3.4;5.4)	3.5	(3.2;3.8)	2.9	(2.3;3.5)	2.9	(2.7;3.1)	4.1	(3.6;4.6)	1.7	(1.7;1.8)
6 months	6.1	(5.2;7.0)	8.4	(6.7;10.0)	6.6	(6.1;7.1)	5.6	(4.7;6.5)	5.3	(4.9;5.6)	7.3	(6.5;8.1)	3.2	(3.1;3.3)
12 months	11.6	(9.9;13.3)	16.3	(12.7;19.8)	12.7	(11.7;13.7)	10.8	(9.1;12.6)	10.0	(9.4;10.7)	14.2	(12.8;15.6)	6.0	(5.8;6.2)

^1^Index contacts due to exacerbation of chronic disease or to new health problem.

^2^Patients with more than one of the five chronic diseases were included in the numerator for each specific disease.

^3^Distribution-related weighted percentage for contacts.

This higher use was also seen in the increased OR for exacerbation at index contact with daytime GP compared to patients without one of the five chronic diseases (Table 5). A significantly higher OR for exacerbation as RFE at index contact was observed for patients with psychiatric disease (OR = 2.15 (95% CI: 1.00; 5.39)) and patients with cancer (OR = 2.17 (95% CI: 1.20; 3.91)) who had more than two daytime contacts during the 30 days prior to the index date (Table 5).

**Table 5:** **Odds Ratio for exacerbation in chronic disease as RFE at the OOH Primary Care Service according to frequency in daytime GP contacts during 30 days prior to the index contact**

			Odds Ratio (95%CI					
Contacts to GP	N (%)		Crude		Adjusted ^1^		Adjusted ^2^	
**Heart disease**	**152**	**(100%)**						
No GP contacts	37	(19.4%)	1		1		1	
1-2 GP contacts	90	(59.4%)	2.19	(1.08;4.46)	2.22	(1.08;4.59)	2.21	(1.47;3.83)
>2 GP contacts	25	(21.2%)	1.41	(0.59;3.41)	1.56	(1.29;3.71)	1.54	(0.51;4.64)
**Lung disease**	**83**	**(100%)**						
No GP contacts	28	(24.6%)	1		1		1	
1-2 GP contacts	41	(52.9%)	2.82	(1.16;6.85)	2.81	(1.15;6.91)	3.20	(1.42;7.17)
>2 GP contacts	14	(22.4%)	1.34	(0.42;4.26)	1.34	(0.42;4.25)	1.49	(1.08;2.05)
**Diabetes**	**381**	**(100%)**						
No GP contacts	109	(24.0%)	1		1		1	
1-2 GP contacts	197	(53.1%)	1.60	(1.06;2.43)	1.55	(1.03;2.36)	1.51	(0.99;2.30)
>2 GP contacts	75	(22.9%)	1.74	(1.02;2.94)	1.56	(0.90;2.70)	1.54	(0.89;2.68)
**Psychiatric disease**	**131**	**(100%)**						
No GP contacts	57	(38.7%)	1		1		1	
1-2 GP contacts	49	(37.3%)	0.99	(0.49;1.99)	0.96	(0.48;1.97)	0.98	(0.48;2.01)
>2 GP contacts	25	(24.0%)	2.38	(0.99;5.71)	2.09	(0.99;5.14)	2.15	(1.00;5.39)
**Cancer**	**336**	**(100%)**						
No GP contacts	103	(23.9%)	1		1		1	
1-2 GP contacts	170	(52.6%)	1.65	(1.07;2.54)	1.61	(1.04;2.50)	1.56	(1.01;2.43)
>2 GP contacts	63	(22.2%)	2.39	(1.37;4.16)	2.19	(1.22;3.94)	2.17	(1.20;3.91)
**2 + diagnose**	**205**	**(100%)**						
No GP contacts	58	(25.4%)	1		1		1	
1-2 GP contacts	104	(52.8%)	1.53	(0.82;2.83)	1.51	(0.82;2.78)	1.49	(0.81;2.76)
>2 GP contacts	43	(21.8%)	1.78	(0.84;3.75)	1.63	(0.75;3.54)	1.63	(0.75;3.56)
**Annual follow-up**			**Crude**		**Adjusted** ^**1**^		**Adjusted** ^**2**^	
**Heart disease**	**152**	**(100%)**						
No annual follow-up	120	(81.2%)	1		1		1	
Annual follow-up	32	(18.8%)	0.82	(0.39;1.73)	0.84	(0.40;1.78)	0.83	(0.39;1.75)
**Lung disease**	**83**	**(100%)**						
No annual follow-up	71	(80.7%)	1		1		1	
Annual follow-up	12	(19.3%)	0.71	(0.21;2.41)	0.70	(0.20;2.48)	0.68	(0.19;2.43)
**Diabetes**	**381**	**(100%)**						
No annual follow-up	284	(73.2%)	1		1		1	
Annual follow-up	97	(26.8%)	1.07	(0.71;1.62)	1.0	(0.65;1.51)	0.98	(0.66;1.55)
**Psychiatric disease**	**131**	**(100%)**						
No annual follow-up	115	(88.0%)	1		1		1	
Annual follow-up	16	(12.0%)	0.59	(0.20;1.74)	0.48	(0.17;1.40)	0.42	(0.14;1.25)
**Cancer**	336	**(100%)**						
No annual follow-up	280	(82.8%)	1		1		1	
Annual follow-up	56	(17.2%)	1.00	(0.58;1.71)	0.92	(0.53;1.58)	0.91	(0.52;1.58)
**2 + diagnose**	**205**	**(100%)**						
No annual follow-up	154	(78.9%)	1		1		1	
Annual follow-up	51	(21.1%)	0.83	(0.62;1.12)	0.63	(0.34;1.17)	0.61	(0.33;1.13)

^1^Adjusted for gender and age.

^2^Ajusted for contact type in the LV-KOS.

For most of the disease groups, the results indicated that patients who had received an annual follow-up had a lower OR for exacerbation at index contact than other patients, although this association did not reach statistical significance. This trend was most evident for patients with lung disease (OR = 0.68 (95% CI: 0.19; 2.43)), patients with psychiatric disease (OR = 0.42 (95% CI: 0.14; 1.25)), and patients with at least two of the five selected chronic diseases (OR = 0.61 (95% CI: 0.33; 1.13)) (Table 5).

## Discussion

### Main findings

Approximately one third of the study population who had at least one face-to-face contact with the daytime GP during the preceding 30 days had contacted the OOH services due to an exacerbation of a chronic disease; a little less for patients with diabetes or cancer. Use of daytime GP was highly associated with OOH contacts due to exacerbation of chronic disease. Except for patients with diabetes, this association were less likely initiated by patients with at least two of the five chronic diseases who had received an annual daytime follow-up consultation during the 12-month period prior to the OOH contact.

### Strengths and limitations

A major strength of this study was the data obtained from the LV-KOS survey. Both the participating GPs and the registered contacts to OOH were highly representative of the organisation and for the contacts provided at the OOH services in the region during the study period [10]. For each OOH contact, the GP noted whether it was due to an exacerbation of an existing chronic disease or due to a new health problem. No previous studies or other data sources provide such specific data on contacts to the OOH services. Furthermore, the identification of patients with the five chronic diseases in question was achieved with high precision and validity by using the unique Danish CPR number as link between the LV-KOS population and the Danish national registries [23–25].

The LV-KOS contacts revealed no exhaustive information as to whether the registered RFE was related to the chronic diseases identified for the patient in our study or due to a competing chronic disease which was unknown to us. This could lead to misclassification for some patients and thus overestimation of the number of exacerbations for the specific disease. We chose to analyse the five frequently occurring chronic diseases separately although this implied potential implications of comorbidity. To explore the impact of multiple diseases on the outcome, we performed several analyses on the group of patients with at least two of the selected chronic diseases, and these results were comparable to the results obtained for the five disease groups.

### Comparison with other studies

Activities in the OOH and medical assessments of RFEs for patients with chronic diseases are poorly described in the literature. However, in a recent retrospective study, Adam et al suggested poor pain management to be the overall dominating RFE with the OOH services among patients with cancer [26]. In our study we found however that patients with cancer who recently consulted the daytime GP more often contacted the OOH services due to a new health problem than patients with other chronic diseases; this finding may indicate a reasonably good overall cancer pain control.

A Norwegian study of contacts to general practice for patients with psychiatric disease found that patients in contact with the OOH Primary Care Service also regularly consulted the daytime GP [27]. The study also concluded that patients seen in out-of-hours care had a high prevalence of severe psychiatric disease symptoms. These results highly correspond with our findings. Our results showed that the OR for exacerbation at the index contact was twice as high for patients with psychiatric disease who had more than two consultations at the daytime GP than for patients with none or 1-2 contacts. This may indicate that patients with psychiatric disease who frequently attend the daytime GP could be the most ill if the exacerbation is regarded as a proxy for the state of health. This may call for increased attention on this group of patients during daytime.

Our study indicates that patients with the five selected chronic diseases are less likely to have an exacerbation at the index contact than other patients, provided that the chronically diseased patients had received an annual follow-up consultation. These findings are in line with the objectives stated in the general guidelines on follow-up consultations for chronic disease cuix. However, despite the proportion of patients with diabetes who had an annual follow-up, the weighted and adjusted estimates showed only minor impact on the OR for exacerbation incidence rates. Despite the specific recommendations on annual follow-up for diabetes and heart disease [28, 29], patients in these two groups had the highest OR for exacerbation at the index contact.

## Conclusions

In a random sample of contacts to the OOH, we found that patients who contacted the OOH due to an exacerbation of a chronic disease had often recently been examined by a daytime GP. We also found an association between frequent use of daytime general practice and use of OOH services due to an exacerbation of chronic disease. This corresponds to the hypothesis that regular OOH attenders present the most severely chronically diseased patients with both high use of daytime GP and high use of OOH health-care services. We could not verify our hypothesis that frequent use of daytime GP might prevent exacerbations as reasons for encounter in the OOH services. We did, however, find a tendency towards fewer OOH exacerbation contacts for patients who had a preventive annual follow-up for their chronic disease. More research is needed to elaborate whether the need for OOH care could be lowered by shared files between daytime GP and the OOH service which is not possible at present. More research is also needed to explore the specific daytime efforts in chronic care that is taken by the GPs and of the patient involvement.

The field needs to be further researched as these findings may indicate a possibility for providing better proactive prevention of acute exacerbations for patients who are currently in need of extensive medical attention from the OOH Primary Care Service.
